# Supplementary material for: CP2 skyrmions and skyrmion crystals in realistic quantum magnets
Source: Nat Commun. 2023 Jun 19;14:3626. doi: 10.1038/s41467-023-39232-8 (PMC10279712; doi:10.1038/s41467-023-39232-8)
Supplement: Supplementary file 1 — Supplemental Information [file 41467_2023_39232_MOESM1_ESM.pdf]

Supplemental Information for "CP^2 Skyrmions and Skyrmion Crystals in Realistic Quantum Magnets"

I. CHARACTERIZATION OF PHASES

In the main text, we present detailed discussions for the two skyrmion crystal phases (SkX-I and SkX-II) that emerge from the classical SU(3) limit of the model Hamiltonian [see Eq. (12) of the main text]. Here we characterize the remaining phases reported in Fig. 1 of the main text.

A. Quantum paramagnet phase and fully-polarized state

The classical limit of the quantum paramagnet (QPM) is described by the coherent state $|\boldsymbol{Z}_j\rangle = |0\rangle_j$, for which all three dipolar components of the color field vanish: $\langle \boldsymbol{Z}_j|\hat{S}_j^{x,y,z}|\boldsymbol{Z}_j\rangle = 0$. However, the remaining five nematic components of the color field may take non-zero values, e.g. $\langle \boldsymbol{Z}_j|\hat{T}_j^8|\boldsymbol{Z}_j\rangle = 1/\sqrt{3}$. In contrast, the fully-polarized state is described by the coherent state $|\boldsymbol{Z}_j\rangle = |1\rangle_j$ that maximizes the dipolar moment along the z axis: $\langle \boldsymbol{Z}_j|\hat{S}_j^z|\boldsymbol{Z}_j\rangle = 1$.

B. Single-$\boldsymbol{Q}$ orderings

A large region of the phase diagram is occupied by the canted spiral (CS) phase described by the coherent state

$$|\boldsymbol{Z}_j\rangle = \cos\theta|0\rangle + e^{i\boldsymbol{Q}\cdot\boldsymbol{r}_j}\sin\theta\cos\phi|1\rangle + e^{-i\boldsymbol{Q}\cdot\boldsymbol{r}_j}\sin\theta\sin\phi|\bar{1}\rangle, \tag{S1}$$

where θ and ϕ are variational parameters and $\boldsymbol{Q}$ is the ordering wave vector. Note that the CS phase has an uniform value of $\langle \hat{S}_j^z\rangle$ for all sites [see Fig. S1**c**]. For a fixed external magnetic field h, the dipole moment of each site, $\langle \boldsymbol{S}_j\rangle$, is continuously suppressed to zero at

$$D_c = h\sqrt{1-\frac{4J^2(\boldsymbol{Q})}{h^2+4J^2(\boldsymbol{Q})}} - 2J(\boldsymbol{Q})\left(1-\frac{2J(\boldsymbol{Q})}{\sqrt{h^2+4J^2(\boldsymbol{Q})}}\right), \tag{S2}$$

that signals the second-order transition into the QPM phase.

Between the Skyrmion crystal phases are two modulated vertical spiral phases, MVS-I and MVS-II, with a polarization plane parallel to the $\hat{z}$-axis. Unlike the case of spirals described by SU(2) coherent states, the magnitude of the dipole moment is continuously modulated as the dipole moment rotates along the $\hat{z}$ axis [see Fig. S1**a b**]. In the large-$D/|J_1|$ limit, the two spirals become vertical spiral states in the pseudo-spin variables.

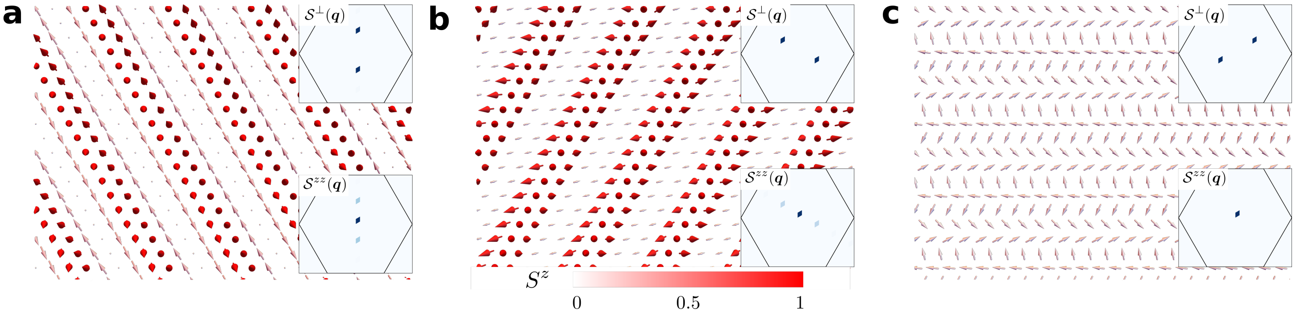

Figure S1. Real space distribution of the dipolar sector of the color fields for the three single-$\boldsymbol{Q}$ orderings. **a** MVS-I. **b** MVS-II. **c** CS. The length of the arrow represents the magnitude of the dipole moment of the color field $|\langle\hat{\boldsymbol{S}}_j\rangle| = \sqrt{(n_j^7)^2+(n_j^5)^2+(n_j^2)^2}$. The color scale of the arrows indicates $\langle\hat{S}_j^z\rangle = -n_j^2$. The insets display the static spin structure factors $\mathcal{S}^{\perp}(\boldsymbol{q}) = \langle n_{\boldsymbol{q}}^7 n_{\bar{\boldsymbol{q}}}^7 + n_{\boldsymbol{q}}^5 n_{\bar{\boldsymbol{q}}}^5\rangle$ and $\mathcal{S}^{zz}(\boldsymbol{q}) = \langle n_{\boldsymbol{q}}^2 n_{\bar{\boldsymbol{q}}}^2\rangle$, with $\boldsymbol{n}_{\boldsymbol{q}} = \sum_j e^{i\boldsymbol{q}\cdot\boldsymbol{r}_j}\boldsymbol{n}_j/\sqrt{N}$.

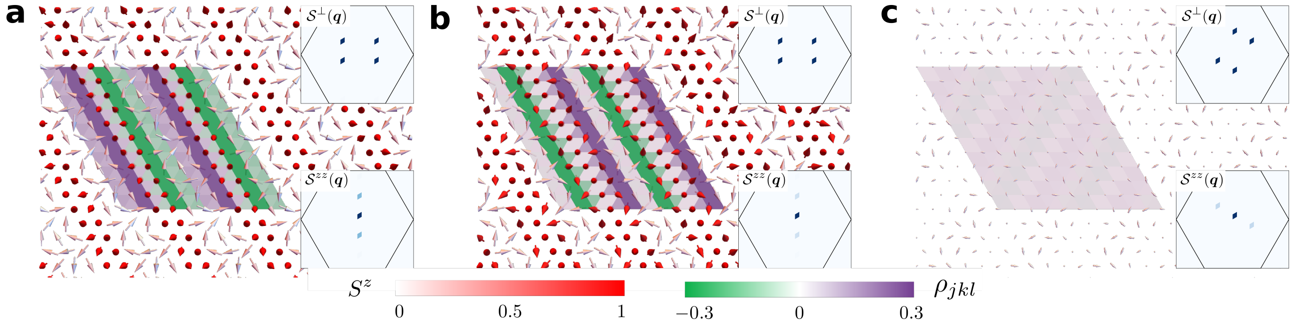

Figure S2. Real space distribution of the dipolar sector of the color fields for the three modulated double-$\boldsymbol{Q}$ orderings: **a** MDQ-I, **b** MDQ-II, and **c** MDQ-III. The length of the arrow represents the magnitude of the dipole moment of the color field $|\langle \hat{\boldsymbol{S}}_j \rangle| = \sqrt{(n^7_j)^2 + (n^5_j)^2 + (n^2_j)^2}$. The color scale of the arrows indicates $\langle \hat{S}^z_j \rangle = -n^2_j$. The insets display the static spin structure factors $\mathcal{S}^{\perp}(\boldsymbol{q}) = \langle n^7_{\boldsymbol{q}} n^7_{\bar{\boldsymbol{q}}} + n^5_{\boldsymbol{q}} n^5_{\bar{\boldsymbol{q}}} \rangle$ and $\mathcal{S}^{zz}(\boldsymbol{q}) = \langle n^2_{\boldsymbol{q}} n^2_{\bar{\boldsymbol{q}}} \rangle$, with $\boldsymbol{n}_{\boldsymbol{q}} = \sum_j e^{i\boldsymbol{q}\cdot\boldsymbol{r}_j} \boldsymbol{n}_j / \sqrt{N}$. The CP^2 skyrmion density distribution ρ_{jkl} [see Eq. (16) of the main text] is indicated by the color of the triangular plaquettes in all three panels.

C. Modulated double-$\boldsymbol{Q}$ orderings

There are three different modulated double-$\boldsymbol{Q}$ (MDQ I-III) orderings in the phase diagram [see Fig. S2]. Similar to the relation between MVS-I and MVS-II, the MDQ-I and MDQ-II phases appearing in the small $D/|J_1|$ region have the same symmetry and are separated by a first-order metamagnetic transition. The MDQ-III phase, which occupies a small region above the QPM phase in the large $D/|J_1|$ region, is the pseudospin counterpart of the 2-q' phase reported in Ref. [1]. Since we have chosen a strong enough easy-axis exchange anisotropy Δ [see Eq. (1) of the main text], which translates into an effective single-ion easy-axis anisotropy $\tilde{\mathcal{D}}$ (K term of Ref. [1]) upon taking the long wavelength limit of the effective pseudospin model [see Eq. (22) of the main text], the MDQ-III phase eventually disappears for $D/|J_1| \gtrsim 25$. The existence of the MDQ-III phase for moderately large values of $D/|J_1|$ results from higher order terms in J_{ij}/D not included in the effective pseudospin model (19) of the main text. As shown in Fig. S2, the CP^2 skyrmion charge distribution of these MDQ orderings displays a stripe structure.

D. 3Q spiral orderings

There are three triple-$\boldsymbol{Q}$ spiral orderings [3$\boldsymbol{Q}$S I-III] whose transverse spin structure factor exhibits dominant weight in one of the three ordering wave vectors $\boldsymbol{Q}_\nu$ ($\nu = 1, 2, 3$) [see Fig. S3 **a-c**]. The CP^2 Skyrmion density of these 3$\boldsymbol{Q}$S phases displays a staggered distribution. Upon increasing D for a fixed value of h, the subdominant weights are continuously suppressed for the 3$\boldsymbol{Q}$S-I and 3$\boldsymbol{Q}$S-II states, leading to a second order phase transition into MVS-I, and MVS-II, respectively. The additional characteristic of the 3$\boldsymbol{Q}$S-III state is that the longitudinal spin structure factor has an equal weight on the three ordering wave vectors $\boldsymbol{Q}_\nu$.

II. DETERMINATION OF PHASE BOUNDARIES

Figure S4 shows the magnetic field dependence of the energy per site and the magnetization for $D/|J_1| = 19$. As we explained in **Methods**, our high-efficient gradient-based variational minimization of the classical Hamiltonian allows us to determine the phase boundaries very accurately. We determine the first-order phase boundaries (solid lines in Fig. S4) from the discontinuous change in the magnetization. For the second-order phase boundary (dash line in Fig. S4) separating the $M = 0$ QPM and the MDQ-III phase, we set the phase boundary at the point where the magnetization M becomes larger than 10^{-5}.

III. BERRY PHASE AND SOLID ANGLE IN CP^2

A. Continuum limit

Topological soliton solutions of the classical color field become well-defined in the continuum limit that holds for $\lambda \gg a$, where λ is the characteristic wavelength of the spin configuration and a is the lattice parameter. In this limit, we use a Taylor

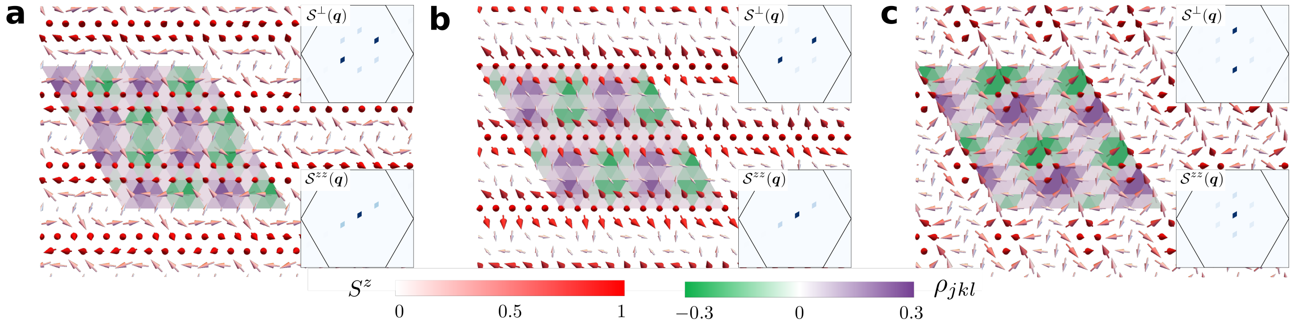

Figure S3. Real space distribution of the dipolar sector of the color fields for the three 3$\boldsymbol{Q}$ spiral orderings: **a** 3$\boldsymbol{Q}$S-I, **b** 3$\boldsymbol{Q}$S-II, and **c** 3$\boldsymbol{Q}$S-III. The length of the arrow represents the magnitude of the dipole moment of the color field $|\langle\hat{\boldsymbol{S}}_j\rangle| = \sqrt{(n_j^7)^2+(n_j^5)^2+(n_j^2)^2}$. The color scale of the arrows indicates $\langle\hat{S}_j^z\rangle = -n_j^2$. The insets display the static spin structure factors $\mathcal{S}^{\perp}(\boldsymbol{q}) = \langle n_{\boldsymbol{q}}^7 n_{\bar{\boldsymbol{q}}}^7 + n_{\boldsymbol{q}}^5 n_{\bar{\boldsymbol{q}}}^5\rangle$ and $\mathcal{S}^{zz}(\boldsymbol{q}) = \langle n_{\boldsymbol{q}}^2 n_{\bar{\boldsymbol{q}}}^2\rangle$, with $\boldsymbol{n}_{\boldsymbol{q}} = \sum_j e^{i\boldsymbol{q}\cdot\boldsymbol{r}_j}\boldsymbol{n}_j/\sqrt{N}$. The CP^2 skyrmion density distribution ρ_{jkl} [see Eq. (16) of the main text] is indicated by the color of the triangular plaquettes in all three panels.

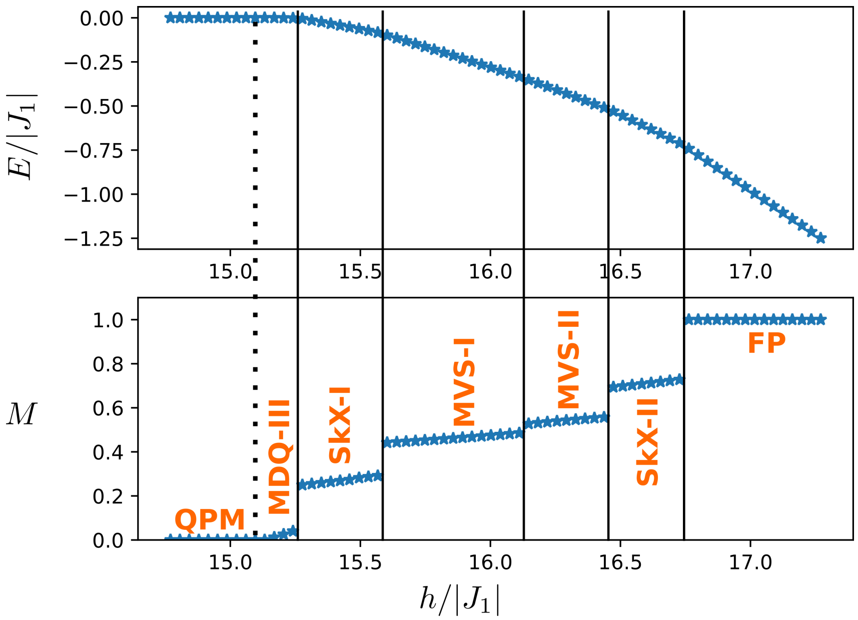

Figure S4. The magnetic field h dependence of the energy per site E (upper panel) and the magnetization M (lower panel) for $D/|J_1| = 19$. The vertical dashed line indicates a second-order phase boundary, and the vertical solid lines mark the first-order phase boundaries.

expansion to express the value of the field n_j^m on the site j (with coordinates $\boldsymbol{r}_j$) in terms of the field n_i^m on the neighboring site located at $\boldsymbol{r}_i$:

$$n_j^m = n_i^m + r_{ij}\partial_k n_i^m + O\left(r_{ij}^2\right) \tag{S3}$$

where $\boldsymbol{r}_j = \boldsymbol{r}_i + r_{ij}\boldsymbol{e}_k, r_{ij} = \left|\boldsymbol{r}_i - \boldsymbol{r}_j\right|$ (r_{ij} coincides with the lattice parameter a when i and j are nearest-neighbor sites) and $\boldsymbol{e}_k$ is a unit vector that points in the k-direction of the plane. Since the lattice spin Hamiltonians that we will consider include only interactions between spins on neighboring sites, we can use Eq. (S3) to define a continuum limit of the spin Hamiltonian by replacing the sum over lattice sites with the integral $a^{-2}\int \mathrm{d}^2x$.

Since the color field takes a constant value n_∞ at spatial infinity for skyrmion configurations, the base plane $\mathbb{R}^2$ can be compactified to S^2. Thus, these spin textures are characterized by the topological degree (or skyrmion charge) of the mapping $\mathfrak{n} : S^2 \mapsto CP^2$:

$$C = -\frac{i}{32\pi}\int \mathrm{d}x\mathrm{d}y\varepsilon_{jk}\,\mathrm{Tr}\left(\mathfrak{n}\left[\partial_j\mathfrak{n}, \partial_k\mathfrak{n}\right]\right) \tag{S4}$$

It is important to note that there is a one-to-one correspondence between the color field $\mathfrak{n}_j$ and $\boldsymbol{Z}_j$ that defines a coherent state. In other words, we can also express the Hamiltonian and the skyrmion density in terms of the $\boldsymbol{Z}_j$ field that in the continuum limit becomes a $\boldsymbol{Z}(\boldsymbol{r})$ field. We can think of both alternative descriptions as the classical limit of the Schrödinger and the Heisenberg representations. In the former case, the dynamical variables are wave functions that become coherent states with coordinates $\boldsymbol{Z}_j$. In the latter case, the dynamical variables are operators $\hat{T}^{\mu}_j$ (observables), which in the classical limit are replaced by their expectation value $\left\langle T^{\mu}_j \right\rangle$ that coincides with the color field n^{μ}_j.

Depending on the application, it may be more convenient to work with one representation of the classical SU(3) spin field or with the other one. For this reason, it is useful to derive an expression of the skyrmion density in terms of the $\boldsymbol{Z}_j$ field. As expected, the skyrmion density defined in Eq. (S4) turns out to be proportional to the Berry curvature of the $\boldsymbol{Z}(\boldsymbol{r})$ field. To demonstrate this statement, we just need to introduce the SU(3) Berry connection,

$$\mathcal{A}(\boldsymbol{r}) = i \langle \mathbf{Z} | \nabla_{\boldsymbol{r}} | \mathbf{Z} \rangle \tag{S5}$$

and the corresponding SU(3) Berry curvature:

$$\mathcal{B}(\boldsymbol{r}) = \nabla \times \mathcal{A}(\boldsymbol{r}). \tag{S6}$$

According to Stokes theorem, the integral of the Berry connection over a closed loop C is equal to the integral of the Berry curvature over the oriented surface enclosed by C:

$$\oint_C \mathcal{A}_j dx^j = \int_{S_C} \left[\partial_x \mathcal{A}_y - \partial_y \mathcal{A}_x\right] \mathrm{d}x\mathrm{d}y. \tag{S7}$$

Our next goal is to demonstrate that the skyrmion density is proportional to the Berry curvature:

$$\partial_x \mathcal{A}_y - \partial_y \mathcal{A}_x = -\frac{i}{16} 2\, \mathrm{Tr}\left(\mathfrak{n}\left[\partial_x \mathfrak{n}, \partial_y \mathfrak{n}\right]\right) = -\frac{i}{16} \varepsilon_{jk}\, \mathrm{Tr}\left(\mathfrak{n}\left[\partial_j \mathfrak{n}, \partial_k \mathfrak{n}\right]\right). \tag{S8}$$

To demonstrate this equivalence, we first need to demonstrate that the infinitesimal "geodesic" SU(3) spin rotation that transforms the coherent state $|\boldsymbol{Z}(\boldsymbol{r})\rangle$ into $|\boldsymbol{Z}(\boldsymbol{r}+\delta\boldsymbol{r})\rangle$ up to a phase factor $e^{i\delta\varphi}$ is:

$$e^{i\delta\varphi} |\mathbf{Z}(\boldsymbol{r}+\delta\boldsymbol{r})\rangle = \hat{U}_{\boldsymbol{r}+\delta\boldsymbol{r},\boldsymbol{r}} |\mathbf{Z}(\boldsymbol{r})\rangle = \left(\mathbb{1} - \frac{i}{4} f_{\mu\nu\eta} n^{\mu} \partial_{\boldsymbol{r}} n^{\nu} \cdot \delta\boldsymbol{r} T^{\eta}\right) |\mathbf{Z}(\boldsymbol{r})\rangle. \tag{S9}$$

In other words,

$$\hat{U}_{\boldsymbol{r}+\delta\boldsymbol{r},\boldsymbol{r}} = \mathbb{1} + i\hat{\boldsymbol{w}} \cdot \delta\boldsymbol{r} = \mathbb{1} - \frac{i}{4} f_{\mu\nu\eta} n^{\mu} \hat{T}^{\eta} \partial_{\boldsymbol{r}} n^{\nu} \cdot \delta\boldsymbol{r}. \tag{S10}$$

To demonstrate this statement, we need to show that the operator field $\mathfrak{n}(\boldsymbol{r})$ is transformed into $\mathfrak{n}(\boldsymbol{r}+\delta\boldsymbol{r})$

$$\begin{aligned} \hat{U}_{\boldsymbol{r}+\delta\boldsymbol{r},\boldsymbol{r}} \mathfrak{n}(\boldsymbol{r}) \hat{U}^{\dagger}_{\boldsymbol{r},\delta\boldsymbol{r}} &= \mathfrak{n}(\boldsymbol{r}) + \frac{i}{4} f_{\mu\nu\eta} n^{\mu} \partial_{\boldsymbol{r}} n^{\nu} \cdot \delta\boldsymbol{r} \left[\mathfrak{n}(\boldsymbol{r}), \hat{T}^{\eta}\right] = \mathfrak{n}(\boldsymbol{r}) + \frac{1}{4} n^{\alpha} n^{\mu} f_{\mu\nu\eta} f_{\alpha\epsilon\eta} \hat{T}^{\epsilon} \partial_{\boldsymbol{r}} n^{\nu} \cdot \delta\boldsymbol{r} \\ &= \mathfrak{n}(\boldsymbol{r}) + \frac{1}{4} n^{\alpha} n^{\mu} \partial_{\boldsymbol{r}} n^{\nu} \cdot \delta\boldsymbol{r} \hat{T}^{\epsilon} \left[\frac{8}{3}\left(\delta_{\mu\alpha}\delta_{\nu\epsilon} - \delta_{\mu\epsilon}\delta_{\alpha\nu}\right) + 4\left(d_{\mu\alpha\eta} d_{\nu\epsilon\eta} - d_{\nu\alpha\eta} d_{\mu\epsilon\eta}\right)\right]. \end{aligned} \tag{S11}$$

By using the following relationships that can be obtained from the constraint given in Eq. (11) of the main text:

$$\partial_{\boldsymbol{r}} n^m n^m = 0, \tag{S12}$$

$$\partial_{\boldsymbol{r}} n^m = 3 d_{mqp} n^p \partial_{\boldsymbol{r}} n^q, \tag{S13}$$

$$d_{\mu\alpha\eta} n^{\mu} n^{\alpha} = \frac{2}{3} n^{\eta}, \tag{S14}$$

$$\frac{2}{3} n^{\eta} d_{\nu\epsilon\eta} \partial_r n^{\nu} = \frac{2}{9} \partial_r n^{\epsilon}, \tag{S15}$$

$$d_{\nu\alpha\eta} \partial_r n^{\nu} n^{\alpha} = \frac{1}{3} \partial_r n^{\eta}, \tag{S16}$$

$$\frac{1}{3} d_{\epsilon\alpha\eta} n^{\mu} \partial_r n^{\eta} = \frac{1}{9} \partial_r n^{\epsilon}, \tag{S17}$$

we obtain the desired result:

$$\hat{U}_{\boldsymbol{r}+\delta\boldsymbol{r},\boldsymbol{r}}\,\mathfrak{n}(\boldsymbol{r})\hat{U}^{\dagger}_{\boldsymbol{r}+\delta\boldsymbol{r},\boldsymbol{r}} = \mathfrak{n}(\boldsymbol{r}) + \partial_{\boldsymbol{r}}\mathfrak{n}(\boldsymbol{r})\cdot\delta\boldsymbol{r} = \mathfrak{n}(\boldsymbol{r}+\delta\boldsymbol{r}). \tag{S18}$$

Since $\partial_{\boldsymbol{r}}|\mathbf{Z}\rangle = i\hat{w}_{\delta\boldsymbol{r}}|\mathbf{Z}\rangle$, we have:

$$\begin{aligned}
\partial_x\mathcal{A}_y - \partial_y\mathcal{A}_x &= i\left(\partial_x\langle\mathbf{Z}|\right)\left(\partial_y|\mathbf{Z}\rangle - \partial_y\langle\mathbf{Z}|\right)\left(\partial_x|\mathbf{Z}\rangle\right) = \\
&= i\left\langle\mathbf{Z}\right|\left[\hat{w}_x,\hat{w}_y\right]\left|\mathbf{Z}\right\rangle - \frac{1}{16}f_{\mu\nu\eta}f_{\eta\gamma\epsilon}f_{\alpha\beta\gamma}n^{\mu}n^{\eta}n^{\epsilon}\partial_x n^{\nu}\partial_y n^{\beta} \\
&= \frac{1}{4}f_{\alpha\beta\gamma}\partial_x n^{\gamma}\partial_y n^{\beta}n^{\alpha} \\
&= -\frac{i}{8}\operatorname{Tr}\left(\mathfrak{n}\left[\partial_x\mathfrak{n},\partial_y\mathfrak{n}\right]\right) = -\frac{i}{16}\varepsilon_{jk}\operatorname{Tr}\left(\mathfrak{n}\left[\partial_j\mathfrak{n},\partial_k\mathfrak{n}\right]\right),
\end{aligned} \tag{S19}$$

where we have used the following relationships:

$$n^{\mu}n^{\epsilon}f_{\mu\nu\eta}f_{\gamma\epsilon\eta} = \frac{8}{3}(n^{\gamma}n^{\nu} - \frac{4}{3}\delta_{\nu\gamma}) + 4(n^{\mu}n^{\epsilon}d_{\mu\gamma\eta}d_{\nu\epsilon\eta} - \frac{2}{3}d_{\nu\gamma\eta}n^{\eta}), \tag{S20}$$

$$\begin{aligned}
\partial_x n^{\nu}n^{\mu}n^{\epsilon}f_{\mu\nu\eta}f_{\gamma\epsilon\eta} &= -\frac{32}{9}\partial_x n^{\gamma} + \frac{4}{3}n^{\mu}d_{\mu\gamma\eta}\partial_x n^{\eta} - \frac{8}{9}\partial_x n^{\gamma} \\
&= -\frac{32}{9}\partial_x n^{\gamma} + \frac{4}{9}\partial_x n^{\gamma} - \frac{8}{9}\partial_x n^{\gamma} = -4\partial_x n^{\gamma}.
\end{aligned} \tag{S21}$$

This concludes the demonstration of Eq. (S8). From this result and Eq. (S4), we obtain:

$$C = -\frac{i}{32\pi}\int \mathrm{d}^2x\varepsilon_{jk}\operatorname{Tr}\left(\mathfrak{n}\left[\partial_j\mathfrak{n},\partial_k\mathfrak{n}\right]\right) = \frac{1}{2\pi}\int \mathrm{d}x\,\mathrm{d}y\left(\partial_x\mathcal{A}_y - \partial_y\mathcal{A}_x\right). \tag{S22}$$

B. On the lattice

The color field is only defined on discrete lattice points for lattice systems. Thus, to compute the skyrmion number of a given spin configuration, we must introduce an interpolation procedure that allows us to define the spin configuration on any point of the plane $\mathbb{R}^2$. This can be done by connecting color fields $\mathfrak{n}_j$ and $\mathfrak{n}_k$ on nearest-neighbor sites j and k along the geodesic in CP^2. According to this prescription, the contribution to the skyrmion number of a given triangular plaquette jkl of the triangular lattice is:

$$\rho_{jkl} = -\frac{i}{32\pi}\int_{\Delta_{jkl}}\mathrm{d}x\mathrm{d}y\varepsilon_{jk}\operatorname{Tr}\left(\mathfrak{n}\left[\partial_j\mathfrak{n},\partial_k\mathfrak{n}\right]\right), \tag{S23}$$

where $\triangle_{jlk}$ is the triangle formed by the lattice sites jkl. Consequently, the total skyrmion number is equal to the sum of this contribution over all the triangles jkl of the triangular lattice:

$$C = \sum_{\Delta_{jkl}}\rho_{jkl}. \tag{S24}$$

Our next step is to demonstrate that:

$$\rho_{jkl} = \frac{1}{2\pi}\left(\gamma_{jl} + \gamma_{lk} + \gamma_{kj}\right), \tag{S25}$$

where

$$\gamma_{kj} = \arg\left[\left\langle\mathbf{Z}_k \mid \mathbf{Z}_j\right\rangle\right] \tag{S26}$$

is the Berry connection on the bond $j \to k$ and

$$\gamma_{jl} + \gamma_{lk} + \gamma_{kj} = \oint_{\Delta_{jkl}}\mathcal{A}_j dx^j \tag{S27}$$

is the Berry phase associated with the triangle jkl. From Eqs. (S7) and (S4), we have

$$\rho_{jkl} = -\frac{i}{32\pi}\int_{\Delta_{jkl}} \mathrm{d}x\mathrm{d}y\varepsilon_{jk}\,\mathrm{Tr}\left(\mathfrak{n}\left[\partial_j\mathfrak{n},\partial_k\mathfrak{n}\right]\right) = \frac{1}{2\pi}\oint_{\Delta_{jkl}} \mathcal{A}_j dx^j. \tag{S28}$$

Consequently, we just need to demonstrate Eq. (S25).

We first note that, *up to a phase factor* $e^{i\delta\varphi}$, the "geodesic" SU(3) spin rotation that connects the coherent states $|\boldsymbol{Z}(\boldsymbol{r})\rangle$ and $|\boldsymbol{Z}(\boldsymbol{r}+\delta\boldsymbol{r})\rangle$ is the one given in Eq. (22), and it can be rewritten as

$$e^{i\delta\varphi}|\boldsymbol{Z}(\boldsymbol{r}+\delta\boldsymbol{r})\rangle = \hat{U}_{\boldsymbol{r},\delta\boldsymbol{r}}|\boldsymbol{Z}(\boldsymbol{r})\rangle = \left(\mathbb{1}+\frac{i}{4}\left[\mathfrak{n}(\boldsymbol{r}),\partial_{\boldsymbol{r}}\mathfrak{n}(\boldsymbol{r})\right]\delta\boldsymbol{r}\right)|\boldsymbol{Z}(\boldsymbol{r})\rangle. \tag{S29}$$

The next observation is that:

$$\mathfrak{n}(\boldsymbol{r})|\boldsymbol{Z}(\boldsymbol{r})\rangle = \frac{2}{\sqrt{3}}|\boldsymbol{Z}(\boldsymbol{r})\rangle \tag{S30}$$

by definition of the coherent state $|\boldsymbol{Z}(\boldsymbol{r})\rangle$. Consequently, we have

$$e^{i\delta\varphi} = \left\langle \boldsymbol{Z}(\boldsymbol{r}+\delta\boldsymbol{r})\middle|\left(\mathbb{1}+\frac{i}{4}\left[\mathfrak{n}(\boldsymbol{r}),\partial_{\boldsymbol{r}}\mathfrak{n}(\boldsymbol{r})\right]\delta\boldsymbol{r}\right)\middle|\boldsymbol{Z}(\boldsymbol{r})\right\rangle \tag{S31}$$

or

$$e^{i\delta\varphi} = \langle \boldsymbol{Z}(\boldsymbol{r}+\delta\boldsymbol{r}) \mid \boldsymbol{Z}(\boldsymbol{r})\rangle + \left\langle \boldsymbol{Z}(\boldsymbol{r})\middle|\left(\frac{i}{4}\left[\mathfrak{n}(\boldsymbol{r}),\partial_{\boldsymbol{r}}\mathfrak{n}(\boldsymbol{r})\right]\delta\boldsymbol{r}\right)\middle|\boldsymbol{Z}(\boldsymbol{r})\right\rangle = \langle \boldsymbol{Z}(\boldsymbol{r}+\delta\boldsymbol{r}) \mid \boldsymbol{Z}(\boldsymbol{r})\rangle, \tag{S32}$$

where we have used that $\langle \boldsymbol{Z}(\boldsymbol{r})|[\mathfrak{n}(\boldsymbol{r}),\partial_{\boldsymbol{r}}\mathfrak{n}(\boldsymbol{r})]|\boldsymbol{Z}(\boldsymbol{r})\rangle = 0$ because of Eq. (S30). This important result shows that to linear order in $\delta\boldsymbol{r}$, the Berry phase accumulated by the rotation of the coherent state $|\boldsymbol{Z}(\boldsymbol{r})\rangle$ along a geodesic of CP^2 is equal the the overlap $\langle \boldsymbol{Z}(\boldsymbol{r}+\delta\boldsymbol{r}) \mid \boldsymbol{Z}(\boldsymbol{r})\rangle$.

Let us consider now the Berry phase that is obtained when the coherent state $\left|\boldsymbol{Z}_j\right\rangle$ is rotated into the coherent state $|\boldsymbol{Z}_k\rangle$ along the SU(3) geodesic that connects these two points. After dividing the rotation $\hat{U}_{\boldsymbol{r}_k,\boldsymbol{r}_j}$ into a product of $N \longrightarrow \infty$ small rotations:

$$\hat{U}_{\boldsymbol{r}_{kj}/N} = \exp\left\{\frac{i}{4N}\left[\mathfrak{n}\left(\boldsymbol{r}_j\right),\mathfrak{n}\left(\boldsymbol{r}_k\right)\right]\right\}, \tag{S33}$$

$$\begin{aligned}
\gamma_{kj} &= \arg\left[\left\langle \boldsymbol{Z}_k\middle|\hat{U}_{\boldsymbol{r}_k,\boldsymbol{r}_j}\middle|\boldsymbol{Z}_j\right\rangle\right] = \arg\left[\left\langle \boldsymbol{Z}_k\middle|\left(\hat{U}_{\boldsymbol{r}_{kj}/N}\right)^N\middle|\boldsymbol{Z}_j\right\rangle\right] \\
&= \arg\left[\left\langle \boldsymbol{Z}_k\middle|\hat{U}_{\boldsymbol{r}_{kj}}/N\middle|\boldsymbol{Z}\left(\boldsymbol{r}_k-\boldsymbol{r}_{kj}/N\right)\right\rangle\left\langle \boldsymbol{Z}\left(\boldsymbol{r}_k-\boldsymbol{r}_{kj}/N\right)\middle|\hat{U}_{\boldsymbol{r}_{kj}/N}\ldots\middle|\boldsymbol{Z}\left(\boldsymbol{r}_j+\boldsymbol{r}_{kj}/N\right)\right\rangle\left\langle \boldsymbol{Z}\left(\boldsymbol{r}_j+\boldsymbol{r}_{kj}/N\right)\middle|\hat{U}_{\boldsymbol{r}_{kj}}/N\middle|\boldsymbol{Z}_j\right\rangle\right],
\end{aligned} \tag{S34}$$

where $\boldsymbol{r}_{kj} \equiv \boldsymbol{r}_k - \boldsymbol{r}_j$ and the last identity is obtained by inserting expansions of the identity between the unitary operations in an orthonormal basis that includes the coherent state $\left|\boldsymbol{Z}\left(\boldsymbol{r}_j+n\boldsymbol{r}_{kj}/N\right)\right\rangle$ for the identity operator that is inserted on the left of $\left(\hat{U}_{\boldsymbol{r}_{kj}/N}\right)^n$ with $1 \le n \le N$. After taking the $N \longrightarrow \infty$ limit and using Eq. (S32),

$$\lim_{N\to\infty}\arg\left[\left\langle \boldsymbol{Z}\left(\boldsymbol{r}_j+(n+1)\boldsymbol{r}_{kj}/N\right)\middle|\hat{U}_{\boldsymbol{r}_{kj}/N}\middle|\boldsymbol{Z}\left(\boldsymbol{r}_j+n\boldsymbol{r}_{kj}/N\right)\right\rangle\right] = \lim_{N\to\infty}\arg\left[\left\langle \boldsymbol{Z}\left(\boldsymbol{r}_j+(n+1)\boldsymbol{r}_{kj}/N\right) \mid \boldsymbol{Z}\left(\boldsymbol{r}_j+n\boldsymbol{r}_{kj}/N\right)\right\rangle\right] \tag{S35}$$

we obtained the desired result:

$$\gamma_{kj} = \arg\left[\left\langle \boldsymbol{Z}_k\middle|\hat{U}_{\boldsymbol{r}_k,\boldsymbol{r}_j}\middle|\boldsymbol{Z}_j\right\rangle\right] = \int_0^{|\boldsymbol{r}_k-\boldsymbol{r}_j|}\langle \boldsymbol{Z}(a)|\partial_a|\boldsymbol{Z}(a)\rangle\,\mathrm{d}a \tag{S36}$$

with $|\boldsymbol{Z}(a)\rangle = \hat{U}_{\boldsymbol{r}_j+a\hat{\boldsymbol{r}}_{kj},\boldsymbol{r}_j}\left|\boldsymbol{Z}\left(\boldsymbol{r}_j\right)\right\rangle$ and $\hat{\boldsymbol{r}}_{kj} \equiv \boldsymbol{r}_k - \boldsymbol{r}_j/\left|\boldsymbol{r}_k-\boldsymbol{r}_j\right|$, which implies Eq. (S27).

IV. MAPPING BETWEEN COLOR FIELD AND SU(3) COHERENT STATES

Eq. (S25) is clearly very useful when we are working with coherent states (the $\boldsymbol{Z}$-field) instead of working with the color field $\mathfrak{n}(\boldsymbol{r})$. In the latter case, it may be useful to find a coherent state $|\boldsymbol{Z}(\boldsymbol{r})\rangle$ associated with the field $\mathfrak{n}(\boldsymbol{r})$ to keep using the simple

formula provided by Eq. (S25). Note that the coherent state $|\boldsymbol{Z}(\boldsymbol{r})\rangle$ is defined up to a phase factor (gauge freedom), which does not affect the value of the Berry phase on a closed loop. Correspondingly, we just need a procedure that allows us to find some state $|\boldsymbol{Z}(\boldsymbol{r})\rangle$ (a particular gauge choice) for a given $\mathfrak{n}(\boldsymbol{r})$.

Eq. (10) of the main text establishes a mapping $\left|\boldsymbol{Z}_j\right\rangle \rightarrow \mathfrak{n}(\boldsymbol{r})$ between an SU(3) coherent state $|\boldsymbol{Z}(\boldsymbol{r})\rangle$ and the color field $\mathfrak{n}(\boldsymbol{r})$. Let us find the inverse mapping $\mathfrak{n}(\boldsymbol{r}) \rightarrow \left|\boldsymbol{Z}_j\right\rangle$ Remember that coherent states are defined up to a phase factor (gauge freedom). Given the highest weight state, $|+1\rangle$ that satisfies:

$$\hat{T}^3_j\,|{+1}\rangle = \frac{2}{\sqrt{3}}\,|{+1}\rangle\,, \tag{S37}$$

we can obtain the coherent state $|\boldsymbol{Z}(\boldsymbol{r})\rangle$ by applying an SU(3) transformation $\hat{U}$ that satisfies

$$\begin{aligned} \left|\boldsymbol{Z}_j\right\rangle &= \hat{U}_j\,|{+1}\rangle \\ \mathfrak{n}_j &= \frac{2}{\sqrt{3}}\hat{U}_j\hat{\hat{T}}^3_j\hat{U}^\dagger_j. \end{aligned} \tag{S38}$$

This immediately implies that $|\boldsymbol{Z}(\boldsymbol{r})\rangle$ is the highest-weight eigenstate of the color field $\mathfrak{n}(\boldsymbol{r})$

$$\mathfrak{n}_j\left|\boldsymbol{Z}_j\right\rangle = \frac{2}{\sqrt{3}}\left|\boldsymbol{Z}_j\right\rangle, \tag{S39}$$

and allows us to obtain the coherent state $|\boldsymbol{Z}(\boldsymbol{r})\rangle$ for given color field $\mathfrak{n}(\boldsymbol{r})$. As expected, the normalized eigenstate $|\boldsymbol{Z}(\boldsymbol{r})\rangle$ is defined up to a multiplicative phase factor.

[1] Leonov, A. O. & Mostovoy, M. Multiply periodic states and isolated skyrmions in an anisotropic frustrated magnet. *Nature Communications* **6**, 8275 (2015). URL https://doi.org/10.1038/ncomms9275.
